# Beta Peak Frequencies at Rest Correlate with Endogenous GABA+/Cr Concentrations in Sensorimotor Cortex Areas

**DOI:** 10.1371/journal.pone.0156829

**Published:** 2016-06-03

**Authors:** Thomas J. Baumgarten, Georg Oeltzschner, Nienke Hoogenboom, Hans-Jörg Wittsack, Alfons Schnitzler, Joachim Lange

**Affiliations:** 1 Institute of Clinical Neuroscience and Medical Psychology, Medical Faculty, Heinrich-Heine-University Düsseldorf, Düsseldorf, Germany; 2 Russell H. Morgan Department of Radiology and Radiological Science, The Johns Hopkins University School of Medicine, Baltimore, Maryland, United States of America; 3 F.M. Kirby Center for Functional Brain Imaging, Kennedy Krieger Institute, Baltimore, Maryland, United States of America; 4 Department of Diagnostic and Interventional Radiology, Medical Faculty, Heinrich-Heine-University Düsseldorf, Düsseldorf, Germany; ARC Centre of Excellence in Cognition and its Disorders (CCD), AUSTRALIA

## Abstract

Neuronal oscillatory activity in the beta band (15–30 Hz) is a prominent signal within the human sensorimotor cortex. Computational modeling and pharmacological modulation studies suggest an influence of GABAergic interneurons on the generation of beta band oscillations. Accordingly, studies in humans have demonstrated a correlation between GABA concentrations and power of beta band oscillations. It remains unclear, however, if GABA concentrations also influence beta peak frequencies and whether this influence is present in the sensorimotor cortex at rest and without pharmacological modulation. In the present study, we investigated the relation between endogenous GABA concentration (measured by magnetic resonance spectroscopy) and beta oscillations (measured by magnetoencephalography) at rest in humans. GABA concentrations and beta band oscillations were measured for left and right sensorimotor and occipital cortex areas. A significant positive linear correlation between GABA concentration and beta peak frequency was found for the left sensorimotor cortex, whereas no significant correlations were found for the right sensorimotor and the occipital cortex. The results show a novel connection between endogenous GABA concentration and beta peak frequency at rest. This finding supports previous results that demonstrated a connection between oscillatory beta activity and pharmacologically modulated GABA concentration in the sensorimotor cortex. Furthermore, the results demonstrate that for a predominantly right-handed sample, the correlation between beta band oscillations and endogenous GABA concentrations is evident only in the left sensorimotor cortex.

## Introduction

Oscillatory activity in the beta (15–30 Hz) frequency range is a prominent signal in the human sensorimotor cortex, both at rest and during motor activity [1–4]. Beta band activity differs across areas and depends on motor output (see [5] for a review). For example, beta band power in sensorimotor cortex decreases during movement, whereas beta band power increases following movement [6].

The majority of studies on beta band activity investigated the role of power (e.g., [7, 8]). In addition to power, there is increasing evidence that beta peak frequency (i.e., the frequency within the beta band with the highest power) is an important and functionally relevant parameter of oscillatory activity [9]. Beta peak frequency differs across distinct recording sites within the sensorimotor cortex [1]. Furthermore, beta peak frequency differs during movement and stimulation of lower and upper limbs, thereby distinguishing between different somatotopic representations [10]. Finally, beta peak frequency seems to be an important factor for the communication between cortical areas and muscles during movement. For example, neuronal activity in the motor cortex and electromyographic activity during movement is coherently coupled at ~20 Hz [11]. This 20 Hz motor cortical activity is thought to optimize motor output by maximal recruitment of motor neurons at a minimum discharge in the pyramidal tract [11].

Animal and modeling studies provide evidence for an essential role of GABAergic interneuronal activity for the generation of beta oscillations in the sensorimotor cortex [12–14]. For example, a study using modeled neuronal networks found increases in the power of beta band oscillations to result from an increase in the synaptic conductance of GABA_A_-mediated inhibition [12]. Further, studies demonstrated increases in human beta power [7, 8, 12, 15, 16] as well as decreases in beta peak frequency [12] (but see [16, 17]) as a result of pharmacological GABAergic modulation. Such modulations of beta power were evident at rest [7, 12] as well as after motor output [8, 15, 17].

While the abovementioned studies demonstrated a causal link between GABA administration and changes in beta band power and peak frequencies, the concentration of GABA and its direct modulation in the sensorimotor cortex was not measured. Thus, the quantitative relation remains unclear. Magnetic resonance spectroscopy (MRS) offers a non-invasive method for in vivo quantification of endogenous neurotransmitter concentrations in spatially restricted cortical regions [18]. While this approach has initially been applied to estimate GABA concentrations especially in occipital cortical areas (e.g., [19, 20]), recent studies also focused on the sensorimotor cortex (e.g., [16, 21, 22]). These studies demonstrated a linear relationship between sensorimotor GABA concentration and post-movement oscillatory beta power. In contrast, no relationship could be demonstrated between sensorimotor GABA concentration and post-movement oscillatory beta peak frequency [16]. Taken together, there are consistent results supporting a general relationship between GABA concentration and beta power in sensorimotor cortex areas. Contrarily, the results concerning beta peak frequency are less consistent. Therefore, the question remains whether beta peak frequency is related to GABA concentrations and if such a potential relation is present at rest (i.e., without movement) and for endogenous (i.e., non-modulated) GABA concentrations.

Here, we investigated whether the peak frequency of ongoing beta band oscillations is correlated to endogenous GABA concentration in the sensorimotor cortex at rest. Beta peak frequencies were determined by magnetoencephalography (MEG) and individual GABA concentrations were measured by means of MRS. Peak frequencies were determined for the left and right sensorimotor cortex, as well as for a control region in the occipital cortex. For these three regions of interest (ROIs), we linearly related peak frequencies to GABA concentrations estimated for analogue cortical areas.

## Materials and Methods

### Subjects

15 subjects (7 male, age: 59.9 ± 9 years (mean ± SD)) participated after providing written informed consent in accordance with the Declaration of Helsinki and the Ethical Committee of the Medical Faculty, Heinrich-Heine-University Düsseldorf. All participants had normal or corrected to normal vision and reported no sensory impairments, known history of neurological disorders or use of neuro-modulatory medication. The subjects were selected from the healthy controls of a sample that was previously reported in [23].

### Behavioral data

Individual handedness was assessed by comparing bi-manual performance (hand dominance test (HDT), [24]). Categorization based on the performance measure resulted in 12 clearly right-handed subjects (HDT score: 29.8 ± 8.1 (mean ± SD)) and 3 subjects with no clear hand preference (HDT score: -6.8 ± 9.7).

### Magnetic resonance spectroscopy (MRS) data

**Fig 1:**
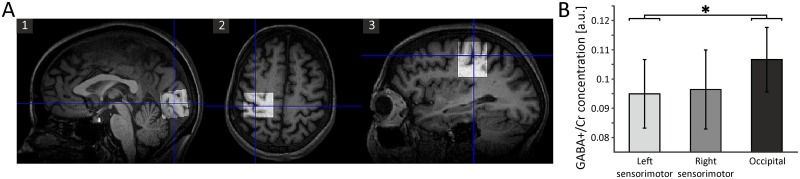
Localization of MRS ROIs and average GABA+/Cr concentrations across MRS ROIs. A) Placement of the occipital voxel in the sagittal plane (1), placement of the left sensorimotor voxel, centered on the hand knob, in the axial (2) and sagittal (3) planes. B) Average GABA+/Cr concentrations for the left and right sensorimotor and occipital MRS ROIs. Error bars represent standard deviations. *: *p* = 0.047.

MRS data were recorded using a 3T whole-body MRI scanner (Siemens MAGNETOM Trio A TIM System, Siemens Healthcare AG, Erlangen, Germany) in connection with a 12-channel head matrix coil. Subjects were instructed to lie in the scanner, relax and refrain from any further activity. For the determination of neurotransmitter concentrations, MRS voxels (3x3x3 cm^3^) were placed in left and right sensorimotor cortices and occipital cortex (Fig 1A). For both sensorimotor cortices, voxels were centered on the respective ‘hand knob’ within the *Gyrus praecentralis* [25], thus covering both motor and somatosensory cortex. The occipital MRS voxel was medially centered on the occipital lobe with the inferior boundary of the voxel aligned with the *Tentorium cerebelli*. For all subjects, voxel placement was performed with the focus to include a maximum portion of cortical volume, as well as a minimal volume of non-cerebral tissues to avoid any additional lipid contamination of the spectra. MRS voxels will be addressed as MRS ROIs (in contrast to MEG ROIs) subsequently.

After the localization of target volumes by means of *T*_*1*_-weighted planning sequences, MEGA-PRESS spectra [26] were acquired (TR = 1500 ms, TE = 68 ms, V = 3x3x3 cm^3^, spectral width = 1200 Hz, 1024 data points). Spectral editing was performed by frequency selective Gaussian refocusing pulses with a bandwidth of 44 Hz. These pulses were irradiated at 1.9 ppm (‘On’ resonance) and 7.5 ppm (‘OFF’ resonance) in alternating fashion. 96 ON and 96 OFF spectra were acquired to give a total of 192 averages (total measurement time: 4.8 minutes per acquisition). Postprocessing and fitting of MEGA-PRESS data was performed with the MATLAB-based tool GANNET 2.0 [27], a software package specifically designed for the analysis of GABA-edited spectra. Postprocessing steps included individual frequency and phase correction of the single acquisitions [28] to reduce potential effects of thermal scanner frequency drift such as linebroadening and subtraction artefacts [29]. Fitting was performed in the frequency domain, with the 3 ppm GABA resonance being modelled as a single Gaussian, and the 3 ppm creatine peak as a single Lorentzian peak. For subsequent analyses, the GABA-to-creatine ratio (GABA+/Cr) was used [30].

GABA+/Cr estimates were not available for every MRS ROI in each subject (see results section for further details). To assess potential differences in GABA+/Cr concentrations across MRS ROIs, GABA+/Cr concentrations were compared across the left, right and occipital MRS ROIs by means of a one-factor repeated-measures ANOVA and post-hoc *t*-tests. To account for the effect of age and handedness, individual values for age and HDT handedness scores were added as covariates to the one-factor repeated-measures ANOVA. In order to ensure that any potential effects would not result from differences in individual cortical grey matter volume across the respective MRS ROIs, we calculated correlations between the individual grey matter volume and the GABA+/Cr concentrations for each MRS ROI, respectively. The rationale of this approach was that, since individual grey matter volume presumably differs across MRS ROIs, it is not feasible to include individual grey matter volume as a covariate in the one-factor repeated-measures ANOVA comparing GABA+/Cr concentrations across MRS ROIs. Although the present approach represents only an indirect control of the influence of individual grey matter volume on GABA+/Cr concentrations, an influence of individual grey matter volume on GABA+/Cr concentration can be deemed implausible if there is no significant correlation between individual grey matter volume and GABA+/Cr concentration in the respective MRS ROI.

### MEG data

#### Experimental design

Subjects were seated in the MEG with all visual stimuli projected on the backside of a translucent screen (60 Hz refresh rate) positioned 57 cm in front of the subjects. Resting-state neuromagnetic activity was recorded during two sessions with a respective duration of 5 minutes, with subjects being instructed to relax and refrain from any additional activity. In the first session, subjects had to focus a dimmed fixation dot (diameter: 0.5 degree) presented in the middle of the translucent screen (eyes open condition (EO)). After completing the first session, subjects were verbally informed regarding the beginning and the instructions of the second session. In the second session, subjects had to close their eyes (eyes closed condition (EC)) but remain awake during the measurement. Stimulus presentation was controlled using Presentation software (Neurobehavioral Systems, Albany, NY, USA).

#### Data recording and preprocessing

Continuous neuromagnetic brain activity was recorded at a sampling rate of 1000 Hz using a 306-channel whole head MEG system (Neuromag Elekta Oy, Helsinki, Finland), including 204 planar gradiometers (102 pairs of orthogonal gradiometers) and 102 magnetometers. Data analysis in the present study was restricted to the planar gradiometers. Electro-oculograms (EOGs) were recorded for offline artifact rejection by applying electrodes above and below the left eye as well as on the outer sides of each eye. Further, an electro-cardiogram (ECG) was recorded for offline artifact rejection by means of two electrodes placed on the left collarbone and the lowest left rib.

Data were offline analyzed using custom-made Matlab (The Mathworks Inc., Natick/MA, USA) scripts and the Matlab-based open source toolbox FieldTrip (http://fieldtriptoolbox.org; [31]). Continuously recorded data were divided into two epochs according to the respective session (EO and EC), starting 3 s after beginning and ending 3 seconds before the end of the respective task. Data were band-pass filtered at 1 Hz to 200 Hz and power line noise was removed by using a band-stop filter encompassing the 50, 100, and 150 Hz components. Data were detrended and the mean of every epoch was subtracted. Continuous data were segmented into trials of 1 s duration with a 0.25 s overlap. Subsequently, trials were semi-automatically and visually inspected for artifacts. Artifacts caused by muscle activity, eye movements or SQUID jumps were removed semi-automatically using a z-score based algorithm implemented in FieldTrip. Excessively noisy channels were removed. To further eliminate cardiac and ocular artifacts, an independent component analysis was computed. Mutual information was calculated between the resulting components and the EOG and ECG channels [32, 33]. Components were sorted according to their level of mutual information and subsequently visually examined regarding their topography and time course. Those components showing high mutual information with EOG and ECG channels as well as topographies and time courses typical for cardiac and ocular artifacts were rejected. Afterwards, removed channels were reconstructed by an interpolation of neighboring channels. After artifact rejection, 292 ± 34.5 (mean ± SD) trials in the EC condition and 304 ± 35.4 trials in the EC condition remained for further analysis. Subsequent analyses were performed separately for the EO and EC condition as well as for a combined data set created by appending the EO and EC condition (EC+EO).

#### Frequency analysis and peak frequency determination

To determine individual peak frequencies, we performed a frequency analysis encompassing all frequencies of the beta band (15 to 30 Hz; [6, 34]) by applying a Fourier transformation over the entire trial duration. Trials were tapered with a single Hanning taper, resulting in a spectral resolution of 1 Hz. Within each condition, spectral power was averaged over all trials for each frequency separately. Power was estimated independently for each of the 204 gradiometers. Subsequently, gradiometer pairs were combined by summing spectral power across the two orthogonal channels, resulting in 102 pairs of gradiometers.

**Fig 2:**
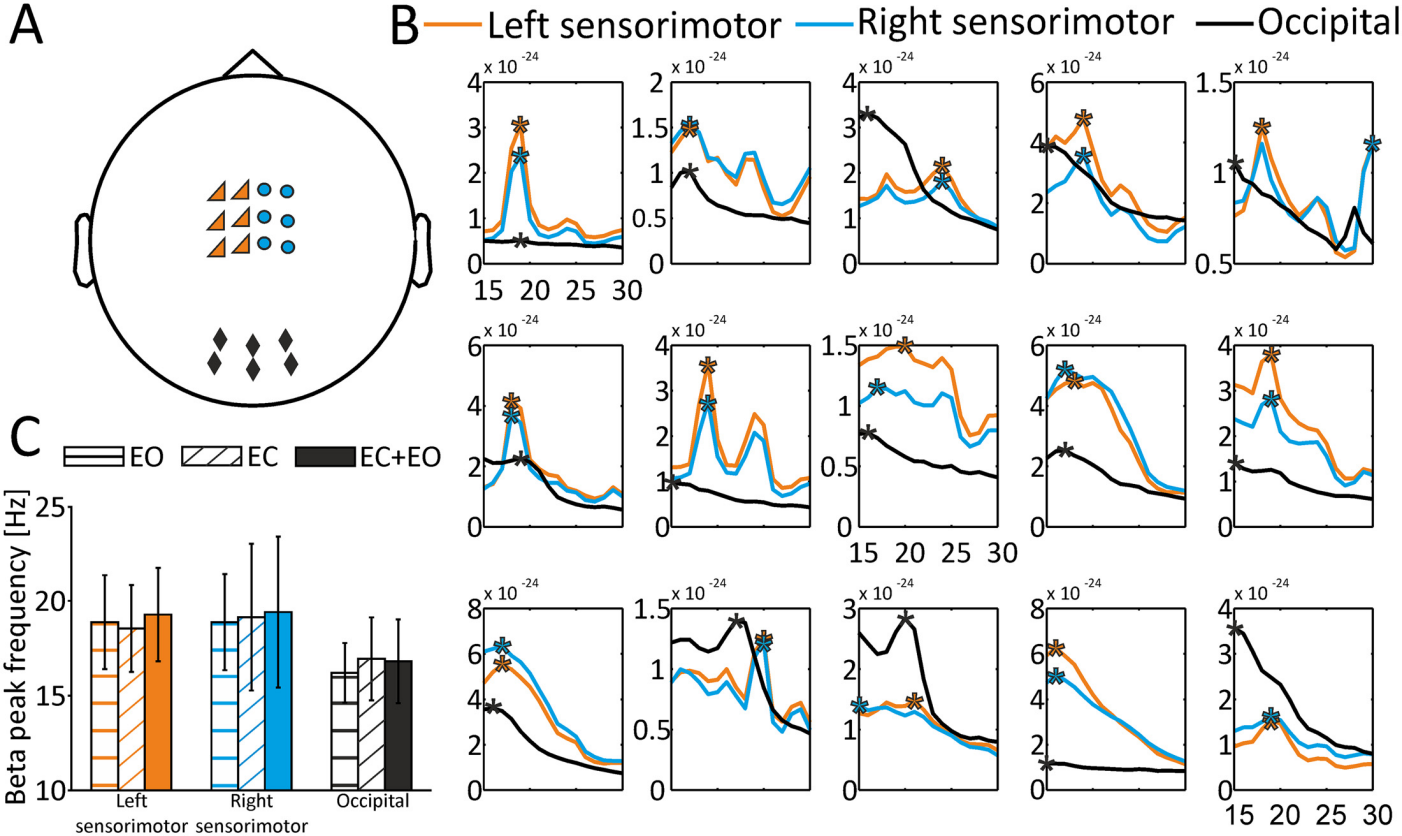
Sensor selection for respective MEG ROIs, individual beta peak frequencies and average beta peak frequencies across MEG ROIs. A) Sensors for left sensorimotor MEG ROI (orange triangles), right sensorimotor MEG ROI (blue dots) and occipital MEG ROI (black diamonds). B) Individual beta peak frequencies for all 15 subjects (EC+EO condition) for left sensorimotor MEG ROI (orange lines), right sensorimotor MEG ROI (blue lines) and occipital MEG ROI (black lines). Individual beta peak frequencies are highlighted by asterisks. C) Average beta peak frequencies separately for all conditions (EO, EC, EC+EO) and all MEG ROIs. Error bars represent standard deviations.

Since GABA-concentrations were assessed for three different MRS ROIs (left and right sensorimotor cortex, occipital cortex; see Fig 1A and methods section (Magnetic resonance spectroscopy (MRS) data) for details), we determined corresponding MEG ROIs by selecting 6 sensor pairs in the left and 6 sensor pairs in the right hemisphere covering the respective sensorimotor cortices (Fig 2A). The selection of sensors was based on previous studies [35, 36]. In addition, we selected 6 posterior sensor pairs covering the occipital cortex [37].

Individual beta peak frequencies were determined semi-automatically within each MEG ROI separately for each subject. For each subject, the frequency showing the maximum power within the predefined beta band (15–30 Hz) was algorithmically selected as the individual peak frequency. Beta peak frequencies were statistically compared between the three MEG ROIs and the three conditions by means of a two-factor repeated-measures ANOVA (main factors: MEG ROI (left sensorimotor, right sensorimotor, occipital) and condition (EO, EC, EC+EO)). Similar to the comparison of GABA+/Cr concentrations, age and HDT handedness scores were included in the analysis as covariates. In case of violations of sphericity, Greenhouse-Geisser corrected values were reported.

To ensure that the peak frequencies determined for the respective sensor selections originate from cortex areas corresponding to the respective MRS ROIs, we additionally computed the respective cortical sources (see S1 Text materials & methods section for details). Subsequently, source level power distributions (S1 and S2 Figs) were visually compared with the location of the MRS ROIs (Fig 1A).

In addition to beta peak frequencies, we performed a control analysis for peak frequencies in the alpha band (8–12 Hz; see S1 Text and S3 and S4 Figs for details on the alpha peak frequency analysis and the corresponding results).

### Correlation of MRS and MEG data

In order to examine the relationship between GABA+/Cr concentrations and resting-state neuromagnetic brain activity, we linearly correlated individual GABA+/Cr concentrations within the respective MRS ROIs with the beta band peak frequencies determined for the corresponding MEG ROIs. We computed correlations (Pearson) within each ROI (e.g., between left sensorimotor MRS ROI and left sensorimotor MEG ROI), thus resulting in 3 correlations for each condition (EO, EC, EC+EO). In addition, we corrected the respective correlations for age, the HDT handedness scores and the individual cortical grey matter volume within the respective MRS ROI by means of partial correlation (Pearson).

## Results

### GABA+/Cr concentrations

GABA+/Cr values were determined in left sensorimotor, right sensorimotor and occipital MRS ROIs (Fig 1). Due to cancellation of the measurements or distorted spectra, GABA+/Cr concentrations could not be estimated for the left sensorimotor, right sensorimotor and occipital MRS ROI in 4, 2, and 1 subjects, respectively (see Table 1 for a summary of GABA+/Cr estimates). For the remaining subjects, a one-factor repeated-measures ANOVA including age and individual HDT handedness scores as covariates yielded a significant difference between GABA+/Cr concentrations in the 3 MRS ROIs (*F*(2,12) = 4.024, *p* = 0.046, 95% CI [left sensorimotor: 0.084, 0.101; right sensorimotor: 0.09, 0.108; occipital: 0.093, 0.112]). Post-hoc *t*-tests revealed significant differences in GABA+/Cr concentration between left sensorimotor and occipital MRS ROIs (*t*(9) = -2.29, *p* = 0.047, 95% CI [-0.019, 0.0001]; Fig 1B). To ensure that any differences between GABA+/Cr concentrations across MRS ROIs did not result from differences in individual cortical grey matter volume, correlations between the individual grey matter volume and the GABA+/Cr concentrations were computed for each MRS ROI. For all three MRS ROIs, no significant correlation between individual grey matter volume and GABA+/Cr concentration could be found (left sensorimotor: *r* = -0.225, *p* = 0.532; right sensorimotor: *r* = -0.112, *p* = 0.729; occipital: *r* = 0.123, *p* = 0.69).

**Table 1:** GABA+/Cr values per MRS ROI.

Subject	GABA+/Cr		
	Left Sensori-motor	Right Sensori-motor	Occipital
1	0.1097	0.1083	0.1054
2	0.0798	0.0713	0.1197
3	0.1035		0.1087
4	0.0995	0.1011	0.1056
5	0.0844	0.0886	0.0940
6		0.0914	0.1213
7		0.0730	0.1134
8		0.1004	0.1166
9			0.1110
10	0.0948	0.1045	0.1073
11	0.0920	0.1187	0.1083
12	0.1078	0.0962	
13	0.1085	0.1014	0.1034
14	0.0781	0.0908	0.0783
15	0.0862	0.1079	0.1000
**Mean**	0.0949	0.0964	0.1066
**SD**	0.0117	0.0135	0.0110

### MEG data

Beta peak frequencies could be determined in all subjects (Fig 2B; Table 2). A two-factor repeated measures ANOVA comparing beta peak frequencies for the factors MEG ROI (left sensorimotor, right sensorimotor, occipital) and condition (EO, EC, EC+EO), with age and individual HDT handedness score included as covariates, yielded no significant main effects for MEG ROI (*F*(2,24) = 0.979, *p* = 0.39, 95% CI [left sensorimotor: 17.591, 20.187, right sensorimotor: 17.473, 20.794, occipital: 15.626, 17.663]) or condition (*F*(2,24) = 1.462, *p* = 0.252, 95% CI [EO: 17.142, 18.813, EC: 17.066, 19.334, EC+EO: 17.365, 19.613]). Likewise, there was no significant interaction between the factors ROI and condition (*F*(1.905, 22.86) = 0.63, *p* = 0.534; Fig 2C). Since no significant results could be found for the factor condition, we chose the combined condition EC+EO for visualization purposes in Fig 2B.

**Table 2:** Beta peak frequencies per MEG ROI and condition.

Beta peak frequency (Hz)									
Subject	Left Sensorimotor			Right Sensorimotor			Occipital		
	EO	EC	ECEO	EO	EC	ECEO	EO	EC	ECEO
1	19	19	19	19	19	19	19	19	19
2	17	17	17	18	16	17	17	17	17
3	24	18	24	24	18	24	15	17	16
4	19	16	19	19	19	19	16	15	15
5	18	18	18	18	30	30	15	15	15
6	18	18	18	18	18	18	17	19	19
7	19	19	19	19	19	19	15	15	15
8	18	19	20	18	17	17	16	15	16
9	17	18	18	17	20	17	17	17	17
10	19	19	19	19	19	19	15	15	15
11	17	17	17	15	17	17	16	16	16
12	25	25	25	25	25	25	15	22	22
13	18	21	21	18	15	15	20	20	20
14	16	15	16	17	15	16	15	17	15
15	19	19	19	19	20	19	15	15	15
**Mean**	18.87	18.53	19.27	18.87	19.13	19.4	16.2	16.93	16.8
**SD**	2.47	2.29	2.46	2.53	3.87	3.98	1.57	2.17	2.21

Source-level analyses of the power distributions for the peak frequencies determined for the left and right sensorimotor MEG ROIs confirmed that the center of activity was centered near the ‘hand knob’ within the *Gyrus praecentralis*, which was selected as the center of the sensorimotor MRS ROIs (see S1 and S2 Figs).

### Correlation of MRS and MEG data

**Fig 3:**
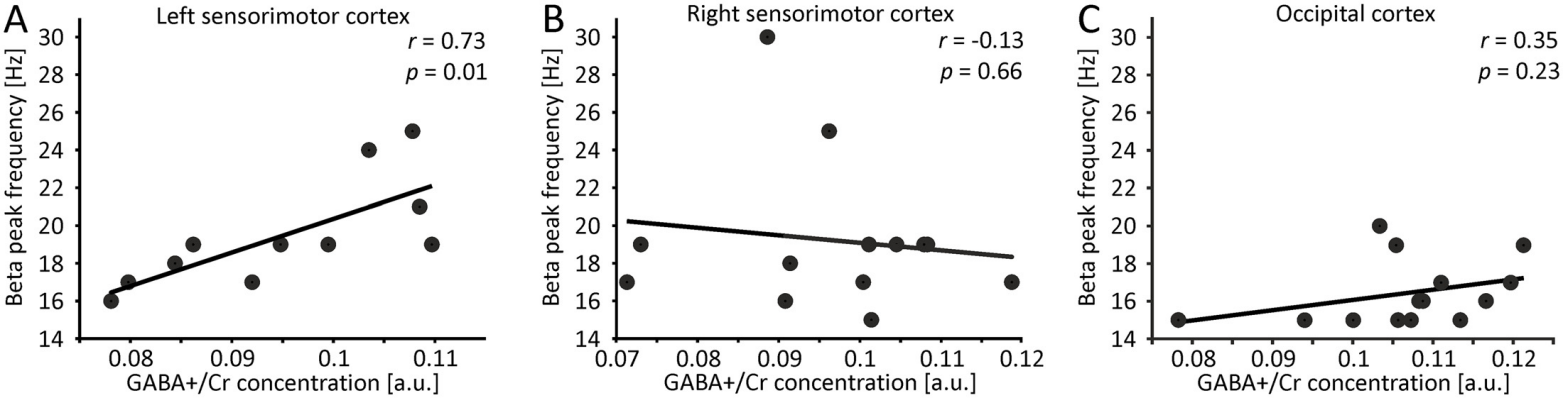
Correlation of beta peak frequencies and GABA+/Cr concentration. (A) Beta peak frequencies calculated for the left sensorimotor MEG ROI and the EC+EO condition correlated with GABA+/Cr estimates from the left sensorimotor MRS ROI. (B) Same as (A), but now for right sensorimotor MEG and MRS ROI. (C) Same as (A), but now for occipital MEG and MRS ROI.

We computed linear correlations between GABA+/Cr concentrations determined in MRS ROIs and beta peak frequencies determined in MEG ROIs, separately for each of the three ROIs (left sensorimotor cortex, right sensorimotor cortex, occipital cortex). Correlation analyses revealed significant linear correlations in the left sensorimotor ROI (EO: *r* = 0.616, *p* = 0.043, EC: *r* = 0.621, *p* = 0.0414, EC+EO: *r* = 0.735, *p* = 0.01; Fig 3A). No significant correlations were found in the right sensorimotor ROI (EO: *r* = -0.139, *p* = 0.65, EC: *r* = -0.067, *p* = 0.827, EC+EO: *r* = -0.134, *p* = 0.662; Fig 3B). Similarly, no significant correlations were found in the occipital ROI (EO: *r* = 0.235, *p* = 0.418, EC: *r* = 0.086, *p* = 0.771, EC+EO: *r* = 0.345, *p* = 0.228; Fig 3C). For all correlations, we additionally partialized out the effect of age, HDT handedness score and respective individual cortical grey matter volume. In line with the uncorrected analyses, corrected correlation analyses revealed significant linear correlations in the left sensorimotor ROI for the EC and the EC+EO condition (EC: *r* = 0.758, *p* = 0.048, EC+EO: *r* = 0.816, *p* = 0.025). For the EO condition, a strong trend could be demonstrated (*r* = 0.724, *p* = 0.066). No significant correlations were found for the right sensorimotor (EO: *r* = -0.139, *p* = 0.721, EC: *r* = -0.084, *p* = 0.829, EC+EO: *r* = -0.108, *p* = 0.783) and occipital cortex (EO: *r* = 0.125, *p* = 0.731, EC: *r* = -0.08, *p* = 0.826, EC+EO: *r* = 0.296, *p* = 0.407).

Since, within each ROI, correlations were highly similar across conditions, we selected the combined condition EC+EO for visualization purposes in Fig 3. Further, correlations within the respective ROIs statistically remained highly similar when correlations were restricted to those subjects for whom valid MRS spectra could be determined for all 3 MRS ROIs (see section MRS data above).

## Discussion

Using magnetoencephalography (MEG) and magnetic resonance spectroscopy (MRS) in healthy human subjects, we investigated the relationship between beta peak frequencies at rest and endogenous (i.e., non-modulated) GABA+/Cr concentrations in the left and right sensorimotor and occipital cortex. The results show significant positive linear correlations between peak frequencies in the beta band (15–30 Hz) and GABA+/Cr concentrations for the left sensorimotor cortex (i.e., higher beta peak frequency was related to a higher GABA+/Cr concentration).

The connection of neuronal oscillatory activity in the beta band and in vivo GABA concentrations has been the topic of various scientific publications. Previous studies that have addressed the general question if sensorimotor beta activity is related to the GABAergic system, applied pharmacological GABAergic modulators [7, 8, 12, 15, 17] and/or investigated movement-related sensorimotor beta activity [8, 15–17]. To our knowledge, the present work is the first study to investigate the connection between beta peak frequency at rest (i.e., without movement or a movement-related task) and non-modulated GABA+/Cr values in the sensorimotor cortex. By focusing exclusively on non-modulated parameters (i.e., no movement-related and pharmaco-induced manipulation), the present study was able to show a correlation between GABA+/Cr concentrations and beta peak frequency at rest.

The analysis of neuromagnetic activity did not demonstrate significant differences in beta peak frequencies across MEG ROIs or conditions. While left and right sensorimotor cortices showed clear peaks in the beta band in all subjects (Fig 2B), beta peaks were less prominent in the occipital cortex, with six subjects showing no clear peak. This is in agreement with the specific role of beta band activity for the sensorimotor cortex [1, 4], while beta band activity in occipital regions is less common. Although the analysis of beta peak frequencies in occipital areas proves to be difficult, we included the occipital MEG ROI as a control condition in order to demonstrate that potential correlations between beta band peak frequency and GABA+/Cr concentrations are not ubiquitously present throughout the cortex, but spatially restricted to sensorimotor cortex areas. Less clear peaks in the beta band for the occipital ROI might be a reason why correlations between GABA+/Cr concentrations and beta peak frequencies were only found for the sensorimotor cortex. This interpretation, however, cannot account for the lack of a significant correlation in right sensorimotor areas, since we found clear peaks in the right sensorimotor cortex for all subjects.

Although the analysis of GABA+/Cr concentrations encompassing all MRS ROIs yielded a significant result, this effect was driven by differences between the left sensorimotor and the occipital MRS ROIs. Since the post-hoc tests showed no significant differences between both sensorimotor MRS ROIs, it is unlikely that hemispherical differences between GABA+/Cr concentrations are responsible for the significant correlation between beta peak frequency and GABA+/Cr concentrations only in the left sensorimotor cortex. Because 12 of 15 subjects in the present study were classified as right-handed, handedness might be an explanation for the unilateral correlation. However, correlations largely remained significant even after correcting for handedness. This finding suggests that handedness alone is unlikely to account for the differences between left and right sensorimotor cortices. Handedness, however, is known to lead to asymmetries with respect to hand representations in the sensorimotor cortex [38–40]. Such asymmetries might lead to regional differences in GABA+/Cr concentration and/or generators of beta frequencies in left and right sensorimotor areas. The results of the correlation analysis further remained virtually unchanged after correcting for age and individual cortical grey matter volume. This excludes the possibility that the unilateral correlation arises as an epiphenomenon due to demographic or neuroanatomical variables. The rather large size of the MRS ROIs poses an additional challenge, since for such voxel sizes it is not possible to separately measure GABA+/Cr concentrations for motor and somatosensory cortex. Although smaller voxel sizes are possible [21], they result in extended measurement time for a comparable signal to noise ratio. Thus, although GABA+/Cr concentrations did not significantly differ between left and right sensorimotor MRS ROIs, our method might have measured more GABA+/Cr concentrations that are unrelated to beta frequency generations in right sensorimotor cortex (i.e., more “noise”). More fined-grained analyses might resolve this problem and shed further light on the relation between GABA concentration and beta peak frequencies. In addition, the sample size of the present study has to be taken into account. Although 15 subjects is a considerable sample size for an MEG/MRS study (i.e., see [12, 16, 19]), an increased sample size would have been preferable. In line with this, it would be interesting to assess both left and right-handed populations of sufficient sample size in future studies to further elucidate the effect of handedness on GABAergic concentrations in sensorimotor cortices.

A general limitation of GABA measurements via MRS is that this method in unable to differentiate between synaptic and extra-synaptic GABA concentrations [22]. Nonetheless, GABA concentrations measured by MRS might primarily reflect extra-cellular GABA concentrations, i.e., the general GABAergic tone [41]. Contrary to intra-cellular GABA concentrations, extra-cellular GABA concentrations would include synaptic concentrations. Beta band oscillations would be primarily related to synaptic GABA concentrations, since this represents the synaptically active neurotransmitter pool [15]. Thus, our results represent correlations with the overall GABA+/Cr concentration of a given voxel, not exclusively for the synaptically active GABA concentration. Despite all potential limitations, we were able to demonstrate a significant positive correlation between GABA+/Cr concentration and beta peak frequency. In addition, various studies using parameters similar to the present study proved that GABA MRS in sensorimotor and occipital cortices yields feasible results (reviewed in [22]). The general feasibility of GABA MRS is further supported by studies that link MRS-derived neurotransmitter concentrations to functional and behavioral measurements [21].

Neuronal oscillations are thought to depend on the balance between excitatory (i.e., glutamatergic synaptic input) and inhibitory (i.e., GABAergic synaptic input) network components [12, 42, 43]. For beta band activity in the sensorimotor cortex, a connection between GABAergic tone and beta band oscillations is supported by studies reporting increases in somatosensory beta band power as an effect of GABAergic modulation by means of positive allosteric GABAergic modulators (e.g., benzodiazepine) [7, 12, 15, 17]. The relation between GABAergic modulation and beta peak frequencies, however, is less clear. While, Jensen and colleagues [12] reported a small decrease (~1.6 Hz) in resting-state beta peak frequency in bilateral sensorimotor cortices after the administration of benzodiazepine, Baker and Baker [17] found no modulation of beta peak frequency after the administration of benzodiazepine. Benzodiazepine is considered to enhance the synaptic GABAergic drive [12]. Simplified, an enhanced GABAergic drive could be related to an increased GABAergic concentration, which would contradict the positive correlation between beta peak frequency and GABA+/Cr levels in the left sensorimotor cortex observed in the present study. Yet, various differences between the studies have to be taken into account. First, Jensen et al. [12] and Baker and Baker [17] measured the influence of pharmacological GABA modulations on beta peak frequencies on the within-subject level. The present study measured non-modulated GABA concentrations and investigated correlations on a between-subject level. Further, while we report a correlation for the left sensorimotor cortex, Jensen and colleagues [12] averaged beta peak frequency over bilateral sensorimotor cortices (thereby not investigating lateral differences). Finally, we measured mostly right-handed subjects, so that an influence of handedness cannot be excluded. The abovementioned studies do not report handedness of their subjects, making a direct comparison difficult.

Gaetz and colleagues [16] found no correlation between beta peak frequency during post-movement beta-rebound and endogenous GABA concentrations for the left motor cortex. Post-movement beta-rebound, however, is intrinsically different from resting state beta activity, as measured in our study. Any differences found between our study and Gaetz et al. [16] might thus be related to different tasks. Taken together, the few existing studies focusing on the connection between beta peak frequency and GABA concentrations in sensorimotor cortex areas strongly vary in experimental setting and assessed parameters, thereby complicating a comparison to our results.

For future studies, it would be interesting to determine how sensorimotor beta peak frequency and GABA concentration both relate on a behavioral level. There is evidence that higher sensorimotor GABA concentrations correlate with slower reaction times in a motor sequence learning task [44]. Here, slower reaction has been interpreted as a result of higher levels of inhibition. Furthermore, higher concentrations of sensorimotor GABA have been related to lower discrimination thresholds in a tactile frequency discrimination task [21]. The authors associated higher GABA concentrations with a potentially higher temporal resolution of tactile perception, which would enable neurons to more closely tune their responses to the stimulus cycles. Such an adjustment of neuronal response to stimulus frequency is considered as the underlying mechanism of the connection between sensorimotor GABA levels and frequency discrimination and to result in lower frequency discrimination thresholds. The influence of oscillatory beta activity on behavioral parameters is less clear. Studies relating individual beta peak frequencies to measures of functional performance apart from motor-related tasks are scarce. Differences in the phase of ongoing beta band oscillations in the somatosensory cortex have been shown to predict the temporal perception of subsequently presented tactile stimuli [45]. Here, the specific beta band frequency showing the biggest phase differences predicted the temporal resolution of tactile perception. Perfetti and colleagues [46] found beta power variations to successfully predict mean reaction time in a visually guided motor task, with a decrease of beta power in left sensory-motor areas corresponding to faster reaction times. In line with this, lower beta-power levels during the time of stimulus presentation were related to a faster reaction towards this stimulus [47]. Taken together, these results suggest an involvement of GABA concentrations and beta band activity within the sensorimotor cortex in the temporal dimension of tactile perception. Thus, further research should investigate if GABA concentration and beta band activity show similar connections to behavioral parameters assessed in parallel.

In conclusion, the present study shows a significant linear correlation between beta peak frequency at rest and non-modulated endogenous GABA concentration measured by spectrally edited MRS. Significant correlations were restricted to the left sensorimotor cortex. While previous studies revealed connections between GABA concentrations and beta band power, our results provide a novel connection between GABA concentrations and peak frequencies in the beta band. In line with previous results from studies using pharmacological modulation of GABA concentrations, these results support a specific role of GABAergic inhibition in the generation of oscillatory beta band activity within the sensorimotor system.

## Supporting Information

S1 Fig Source reconstruction of the average power distribution for individual beta peak frequencies determined for the left sensorimotor MEG ROI. A) Source power projected on the surface of the MNI template brain. The striped square approximates the size and position of the left sensorimotor MRS ROI. B) Source power projected on the sagittal plane of the MNI template brain. Source plots are masked to highlight power maxima.
(TIF)

S2 Fig Source reconstruction of the average power distribution for individual beta peak frequencies determined for the right sensorimotor MEG ROI. A) Source power projected on the surface of the MNI template brain. The striped square approximates the size and position of the left sensorimotor MRS ROI. B) Source power projected on the sagittal plane of the MNI template brain. Source plots are masked to highlight power maxima.
(TIF)

S3 Fig Sensor selection for respective MEG ROIs, individual alpha peak frequencies and average alpha peak frequencies across MEG ROIs. A) Sensors for left sensorimotor MEG ROI (orange triangles), right sensorimotor MEG ROI (blue dots) and occipital MEG ROI (black diamonds). B) Individual alpha peak frequencies for all 15 subjects (EC+EO condition) for left sensorimotor MEG ROI (orange lines), right sensorimotor MEG ROI (blue lines) and occipital MEG ROI (black lines). Individual alpha peak frequencies are highlighted by asterisks. C) Average alpha peak frequencies separately for all conditions (EO, EC, EC+EO) and all MEG ROIs. Error bars represent standard deviations.
(TIF)

S4 Fig Correlation of alpha peak frequencies and GABA+/Cr concentration. (A) Alpha peak frequencies calculated for the left sensorimotor MEG ROI and the EC+EO condition correlated with GABA+/Cr estimates from the left sensorimotor MRS ROI. (B) Same as (A), but now for right sensorimotor MEG and MRS ROI. (C) Same as (A), but now for occipital MEG and MRS ROI.
(TIF)

S1 Text Supporting information materials & methods and results. (DOCX)
