# Supplementary figures and images for: Beta Peak Frequencies at Rest Correlate with Endogenous GABA+/Cr Concentrations in Sensorimotor Cortex Areas
Source: PLoS One. 2016 Jun 3;11(6):e0156829. doi: 10.1371/journal.pone.0156829 (PMC4892568; doi:10.1371/journal.pone.0156829)

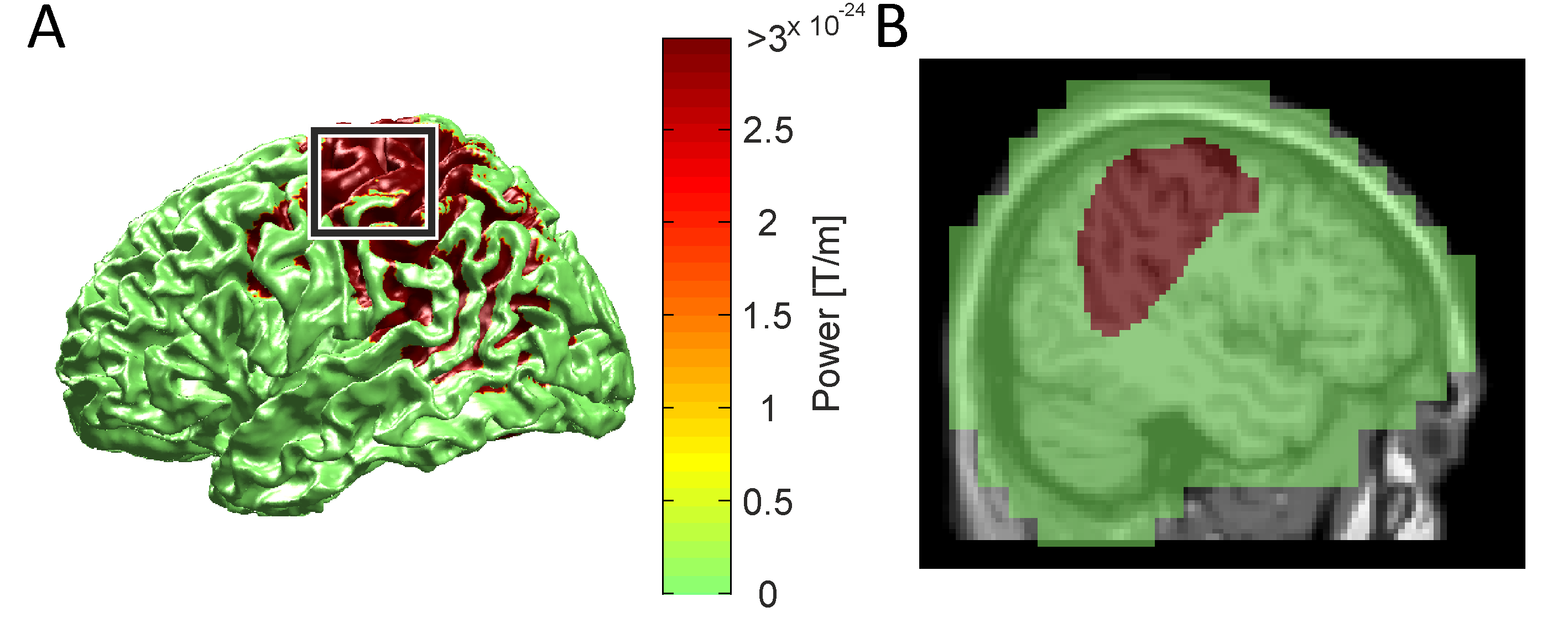

Supplement: S1 Fig — A) Source power projected on the surface of the MNI template brain. The striped square approximates the size and position of the left sensorimotor MRS ROI. B) Source power projected on the sagittal plane of the MNI template brain. Source plots are masked to highlight power maxima. (TIF) [file pone.0156829.s001.tif]

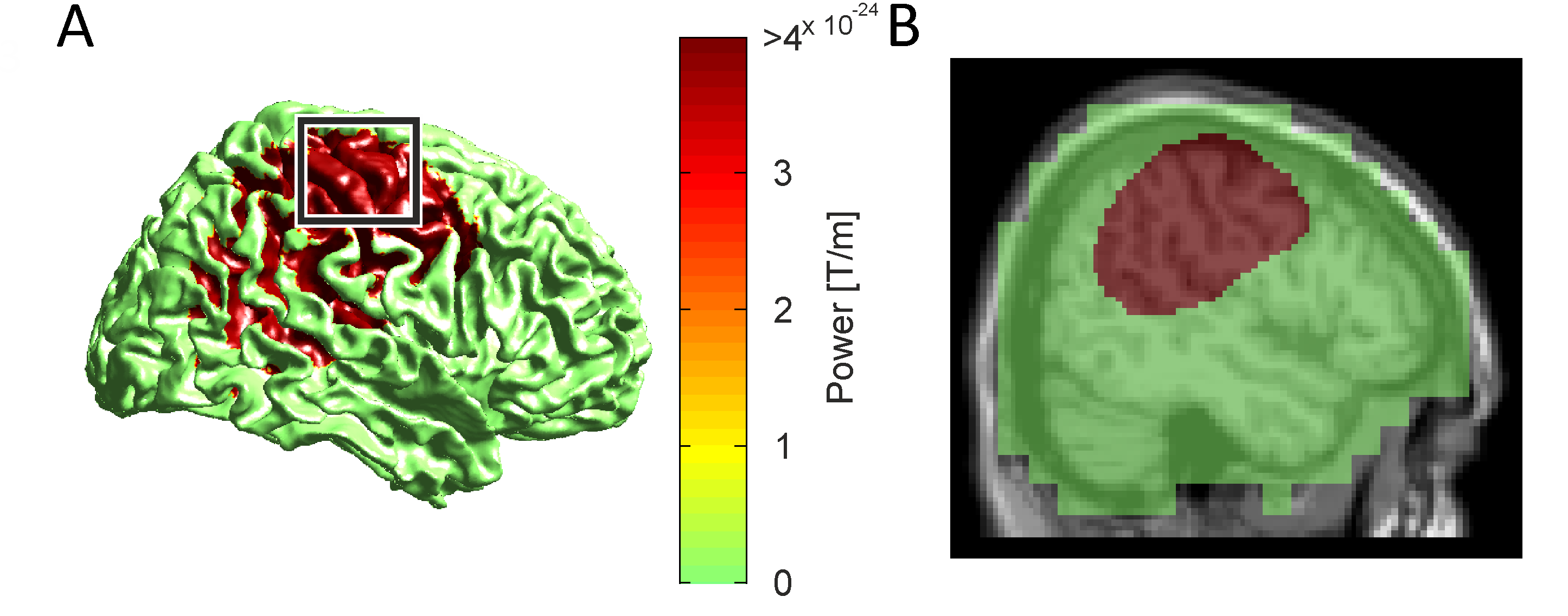

Supplement: S2 Fig — A) Source power projected on the surface of the MNI template brain. The striped square approximates the size and position of the left sensorimotor MRS ROI. B) Source power projected on the sagittal plane of the MNI template brain. Source plots are masked to highlight power maxima. (TIF) [file pone.0156829.s002.tif]

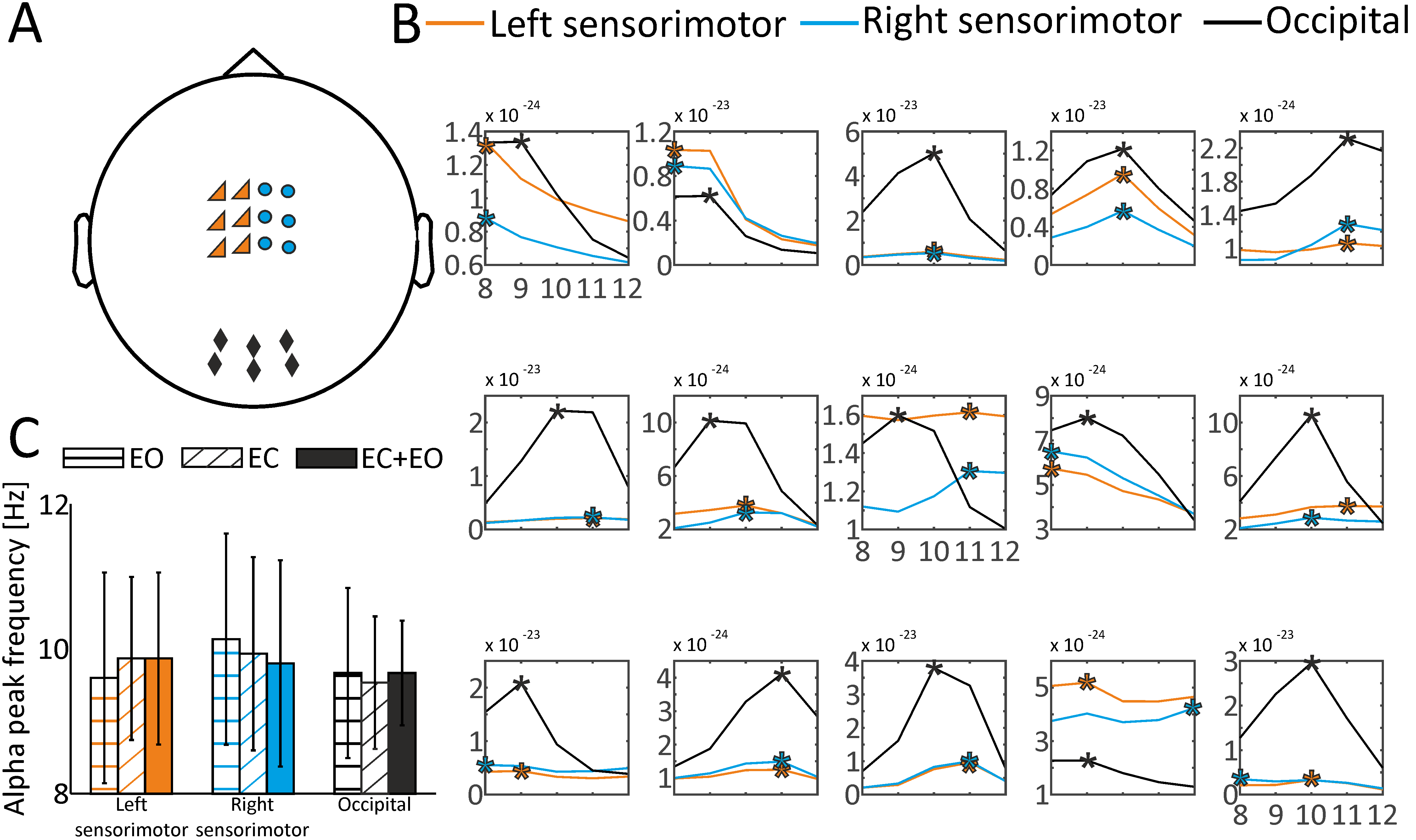

Supplement: S3 Fig — A) Sensors for left sensorimotor MEG ROI (orange triangles), right sensorimotor MEG ROI (blue dots) and occipital MEG ROI (black diamonds). B) Individual alpha peak frequencies for all 15 subjects (EC+EO condition) for left sensorimotor MEG ROI (orange lines), right sensorimotor MEG ROI (blue lines) and occipital MEG ROI (black lines). Individual alpha peak frequencies are highlighted by asterisks. C) Average alpha peak frequencies separately for all conditions (EO, EC, EC+EO) and all MEG ROIs. Error bars represent standard deviations. (TIF) [file pone.0156829.s003.tif]

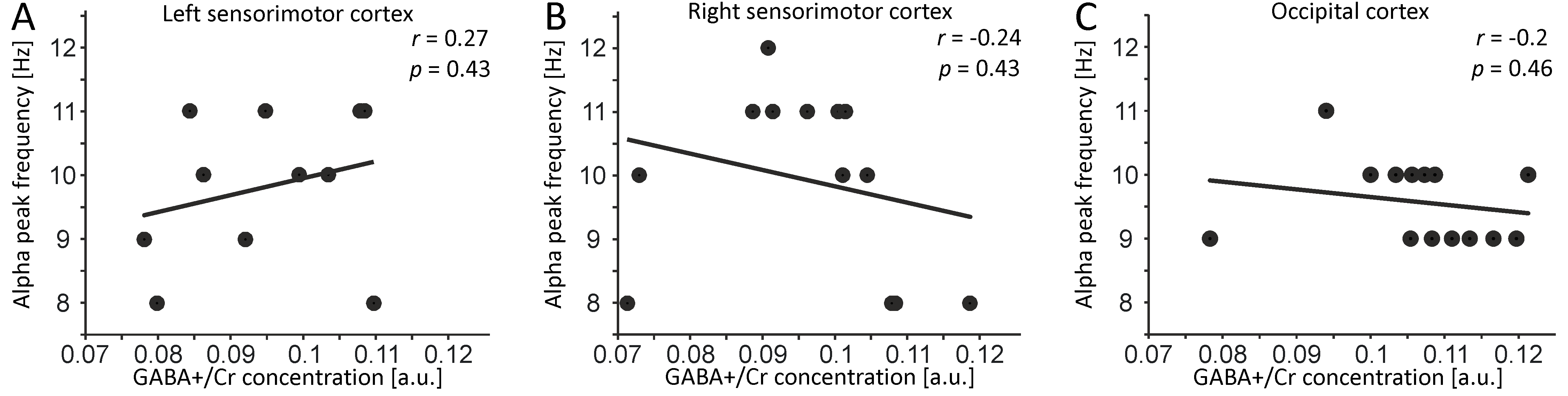

Supplement: S4 Fig — (A) Alpha peak frequencies calculated for the left sensorimotor MEG ROI and the EC+EO condition correlated with GABA+/Cr estimates from the left sensorimotor MRS ROI. (B) Same as (A), but now for right sensorimotor MEG and MRS ROI. (C) Same as (A), but now for occipital MEG and MRS ROI. (TIF) [file pone.0156829.s004.tif]
