# Supplementary material for: Beta Peak Frequencies at Rest Correlate with Endogenous GABA+/Cr Concentrations in Sensorimotor Cortex Areas
Source: PLoS One. 2016 Jun 3;11(6):e0156829. doi: 10.1371/journal.pone.0156829 (PMC4892568; doi:10.1371/journal.pone.0156829)
Supplement: S1 Text — (DOCX) [file pone.0156829.s005.docx]

**Supporting Information**

**Materials & Methods**

**Source-level analysis of sensorimotor peak frequencies**

In order to validate that the peak frequencies measured for the MRS ROIs and GABA+/Cr concentrations measured for the MEG ROIs originate from corresponding cortex areas, we computed the inverse solution for the beta peak frequencies determined for the left and right sensorimotor cortex. Since, for reasons of clarity, we presented the results of the correlation analyses for the combined EO+EC condition, we also selected this combined EO+EC condition for the computation of the inverse solution. To locate the cortical sources of the respective beta peak frequencies determined for the left and right sensorimotor MEG ROIs, we calculated source-level power estimates by means of an adaptive spatial filtering technique (DICS, [48]). A regular 3D grid with 1 cm resolution was applied to the Montreal Neurological Institute (MNI) template brain. Subsequently, individual grids for each subject were computed by linearly warping the structural MRI of each subject onto the MNI template brain and applying the inverse of the warp to the MNI template grid. For two subjects, no individual structural MRI was available. Instead, we used the MNI template brain. A lead-field matrix was computed for each grid point employing a realistically shaped single-shell volume conduction model [49]. The cross-spectral density (CSD) matrix between all MEG gradiometer sensor pairs was computed for the respective individual peak frequency determined for the left and right sensorimotor MEG ROIs. Based on the CSD and the lead-field matrix, common spatial filters for each subject were constructed for each individual grid point. CSD matrices of single trials were projected through those spatial filters, resulting in single-trial estimates of source power [50, 51]. Subsequently, source power estimates were averaged over trials and subjects. The maxima of the group-level source power estimates for peak frequencies determined for the left (S1 Fig) and right sensorimotor cortex (S2 Fig) were displayed on the MNI template brain. Based on these results, the location of the source-level power distributions of the respective peak frequencies were compared with the location of the MRS ROIs.

**Analysis of alpha band peak frequencies**

Oscillatory activity in the beta (15-30 Hz) frequency range is often reported specifically for the sensorimotor cortex [1–4]. However, we also related beta peak frequencies with GABA+/Cr concentrations in the occipital cortex. This served as a control condition in order to show that potential correlations between beta peak frequency and GABA+/Cr concentrations are not ubiquitously present throughout the cortex, but spatially restricted to sensorimotor cortex areas. In addition to beta peak frequencies, we investigated the relationship between GABA+/Cr concentration and alpha peak frequencies. To determine individual alpha peak frequencies, we performed a frequency analysis encompassing all frequencies of the alpha band (8 to 12 Hz) by applying a Fourier transformation over the entire trial duration. Trials were tapered with a single Hanning taper, resulting in a spectral resolution of 1 Hz. Within each condition, spectral power was averaged over all trials for each frequency separately. Power was estimated independently for each of the 204 gradiometers. Subsequently, gradiometer pairs were combined by summing spectral power across the two orthogonal channels, resulting in 102 pairs of gradiometers.

Since GABA-concentrations were assessed for three different MRS ROIs (left and right sensorimotor cortex, occipital cortex; see Fig 1A and methods section (MRS data, Spectroscopy) for details), we determined corresponding MEG ROIs by selecting 6 sensor pairs in the left and 6 sensor pairs in the right hemisphere covering the respective sensorimotor cortices (Fig 2A). The selection of sensors was based on previous studies [36, 52]. In addition, we selected 6 posterior sensor pairs covering the occipital cortex [37].

Individual alpha peak frequencies were determined manually within each MEG ROI separately for each subject. For each subject, the frequency showing the maximum power within the predefined alpha band (8-12 Hz) was selected as the individual peak frequency (S3 Fig). Alpha peak frequencies were statistically compared between the three MEG ROIs and the three conditions by means of a two-factor repeated-measures ANOVA (main factors: MEG ROI (left sensorimotor, right sensorimotor, occipital) and condition (EO, EC, EC+EO)). Similar to the comparison of GABA+/Cr concentrations, age and HDT handedness scores were included in the analysis as covariates. In case of violations of sphericity, Greenhouse-Geisser corrected values were reported.

**Correlation of alpha peak frequencies and MRS data**

In order to examine the relationship between GABA+/Cr concentrations and resting-state neuromagnetic brain activity, we linearly correlated individual GABA+/Cr concentrations within the respective MRS ROIs with the alpha peak frequencies determined for the corresponding MEG ROIs (S4 Fig). We computed correlations (Pearson) within each ROI (e.g., between left sensorimotor MRS ROI and left sensorimotor MEG ROI), thus resulting in 3 correlations for each condition (EO, EC, EC+EO). In addition, we corrected the respective correlations for age, the HDT handedness scores and the individual cortical grey matter volume within the respective MRS ROI by means of partial correlation (Pearson).

**Results**

**Analysis of alpha band peak frequencies**

Alpha peak frequencies were determined in all subjects (S3 Fig). Alpha band peak frequencies were compared by means of a two-factor repeated measures ANOVA for the factors MEG ROI (left sensorimotor, right sensorimotor, occipital) and condition (EO, EC, EC+EO), with age and HDT handedness scores included in the analysis as covariates. The analysis yielded no significant main effect for the factor MEG ROI (*F*(2,24) = 0.896, *p* = 0.421, 95% CI [left sensorimotor: 9.216, 10.339, right sensorimotor: 9.331, 10.58, occipital: 9.147, 10.098]) and condition (*F*(1.115,13.386) = 0.313, *p* = 0.61, 95% CI [EO: 9.121, 10.479, EC: 9.268, 10.288, EC+EO: 9.257, 10.299]). Likewise, there was no significant interaction between the factors ROI and condition (*F*(.1.942, 23.299) = 0.393, *p* = 0.673). Since no significant results could be found for the factor condition, we chose the combined condition EC+EO for visualization purposes in S3 Fig.

**Correlation of alpha band peak frequencies and MRS data**

We computed linear correlations between GABA+/Cr concentrations determined in MRS ROIs and alpha peak frequencies determined in MEG ROIs, separately for each of the three ROIs (left sensorimotor cortex, right sensorimotor cortex, occipital cortex). Correlation analyses revealed no significant linear correlations in the left sensorimotor ROI (EO: *r* = 0.348, *p* = 0.294, EC: *r* = 0.196, *p* = 0.564, EC+EO: *r* = 0.265, *p* = 0.43; S4A Fig). No significant correlations were found in the right sensorimotor ROI (EO: *r* = 0.099, *p* = 0.747, EC: *r* = -0.13, *p* = 0.671, EC+EO: *r* = -0.241, *p* = 0.428; S4B Fig). Similarly, no significant correlations were found in the occipital ROI (EO: *r* = 0.025, *p* = 0.932, EC: *r* = -0.029, *p* = 0.921, EC+EO: *r* = -0.204, *p* = 0.485; S4C Fig). In addition, we partialized out the effect of age, HDT handedness score and respective individual cortical grey matter volume. Correlations remained non-significant after taking these factors into account (left sensorimotor ROI: EO: *r* = 0.375, *p* = 0.408, EC: *r* = 0.219, *p* = 0.637, EC+EO: *r* = 0.299, *p* = 0.515; right sensorimotor ROI: EO: *r* = -0.217, *p* = 0.574, EC: *r* = -0.387, *p* = 0.303, EC+EO: *r* = -0.509, *p* = 0.161, occipital cortex: EO: *r* = -0.044, *p* = 0.904, EC: *r* = -0.109, *p* = 0.763, EC+EO: *r* = -0.365, *p* = 0.299). Since, within each ROI, correlations were highly similar across conditions, we selected the combined condition EC+EO for visualization purposes in S4 Fig.

**References**

48. Gross J, Kujala J, Hamalainen M, Timmermann L, Schnitzler A, Salmelin R. Dynamic imaging of coherent sources: Studying neural interactions in the human brain. Proceedings of the National Academy of Sciences. 2001; 98:694–99.

49. Nolte G. The magnetic lead field theorem in the quasi-static approximation and its use for magnetoencephalography forward calculation in realistic volume conductors. Physics in Medicine and Biology. 2003; 48:3637.

50. Hoogenboom N, Schoffelen J, Oostenveld R, Fries P. Visually induced gamma-band activity predicts speed of change detection in humans. NeuroImage. 2010; 51:1162–67.

51. Baumgarten TJ, Schnitzler A, Lange J. Prestimulus alpha power influences tactile temporal perceptual discrimination and confidence in decisions. Cerebral Cortex. 2016; 26:891-903

52. van Ede F, Lange FP de, Maris E. Anticipation increases tactile stimulus processing in the ipsilateral primary somatosensory cortex. Cerebral Cortex. 2014; 24:2562–71.
